# Defensins as a promising class of tick antimicrobial peptides: a scoping review

**DOI:** 10.1186/s40249-022-00996-8

**Published:** 2022-06-20

**Authors:** Jiahui Wu, Xia Zhou, Qiaoqiao Chen, Zhiqiang Chen, Jinyu Zhang, Lele Yang, Yuxuan Sun, Guohui Wang, Jianfeng Dai, Tingting Feng

**Affiliations:** 1 Institute of Biology and Medical Sciences, Jiangsu Key Laboratory of Infection and Immunity, Soochow University, Suzhou, China; 2 School of Biology and Basic Medical Science, Suzhou Medical College of Soochow University, Suzhou, China; 3 Department of Nuclear Medicine, The First Affiliated Hospital of Soochow University, Suzhou, China; 4 School of Life Science and Technology, Weifang Medical University, Weifang, China

**Keywords:** Tick, Antimicrobial peptide, Defensin, Antimicrobial activity

## Abstract

**Background:**

Ticks are hematophagous parasites that transmit an extensive range of pathogens to their vertebrate hosts. Ticks can destroy invading microorganisms or alleviate infection via their rudimentary but orchestrated innate immune system. Antimicrobial peptides (AMPs) are important components of tick innate immunity. Among these humoral effector molecules, defensins are well-studied and widely identified in various species of Ixodidae (hard ticks) and Argasidae (soft ticks). This review was aimed at presenting the characterization of tick defensins from structure-based taxonomic status to antimicrobial function.

**Main text:**

All published papers written in English from 2001 to May 2022 were searched through PubMed and Web of Science databases with the combination of relevant terms on tick defensins. Reports on identification and characterization of tick defensins were included. Of the 329 entries retrieved, 57 articles were finally eligible for our scoping review.

Tick defensins mainly belong to the antibacterial ancient invertebrate-type defensins of the *cis*-defensins superfamily. They are generally small, cationic, and amphipathic, with six cysteine residues forming three intra-molecular disulfide bonds. Tick defensins primarily target membranes of a variety of pathogens, including Gram-positive and Gram-negative bacteria, fungi, viruses, and protozoa. Since tick defensins have a high degree of variability, we summarize their common biological properties and enumerate representative peptides. Along with the various and potent antimicrobial activities, the role of tick defensins in determining vector competence is discussed.

**Conclusions:**

Due to their broad-spectrum antimicrobial activities, tick defensins are considered novel candidates or targets for controlling infectious diseases.

**Graphical Abstract:**

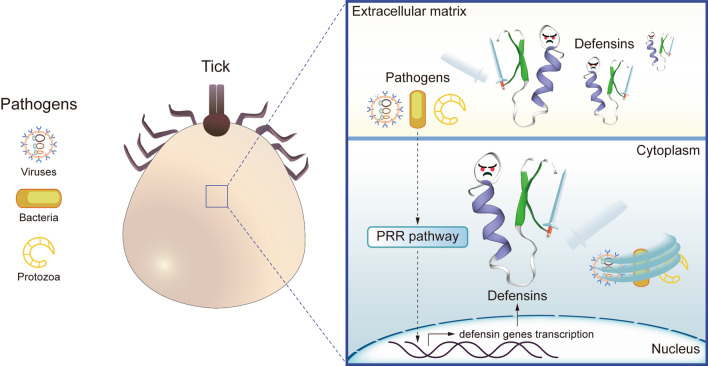

## Background

Ticks are ectoparasitic arthropods that obligatorily suck blood from vertebrate hosts [1]. To date, more than 950 tick species have been identified, which are mainly divided into two families: Ixodidae (hard ticks) and Argasidae (soft ticks) [2, 3]. Ticks are second only to mosquitoes as vectors of pathogens in humans and can transmit multiple pathogens comprising bacteria, fungi, viruses, protozoa, and nematodes. The tick-borne diseases, such as Lyme disease, tick-borne encephalitis, human granulocytic anaplasmosis, rickettsioses, babesiosis, and African swine fever, pose an increasing threat to public health and bring enormous economic losses to livestock production globally [4].

The pathogens acquired within the blood meal from infected hosts must evade tick immune responses on their route through the tick body. The initial organ is the tick midgut, where the pathogen interacts with resident microbiota and cytotoxic molecules. Then the infectious agent colonizes to the gut epithelium followed by migrating to the salivary glands through hemocoel filled with hemolymph. Notably, the tick salivary glands produce saliva to return excess water and ions to the host for concentrating the blood meal. Thus, the tick saliva is a vehicle for the pathogen to be successfully transmitted to the next host in the subsequent feeding stage. Moreover, the saliva provides various bioactive factors which modulate host hemostasis and immune responses, facilitating pathogen acquisition. Some microorganisms can also infect tick ovaries and thereby gain access to progeny through transovarial transmission [5, 6].

Like other invertebrates, ticks defend themselves with a primitive innate immune system consisting of humoral and cellular responses. The cell-mediated action is represented by phagocytosis, encapsulation, and nodulation; while the humoral defense depends on a variety of humoral factors such as pattern recognition receptors, complement-related molecules, lectins, and antimicrobial peptides (AMPs) [7]. The well-studied and best-known AMP is defensin, a potent effector element of innate immunity. Defensins are ubiquitously expressed in a wide range of eukaryotic organisms involving vertebrates, invertebrates, plants, and fungi. They are deeply diverse in terms of sequence but are mostly small (less than 10 kDa), cysteine-rich, cationic, and amphipathic [8]. It has been over two decades since tick defensins were firstly reported in the soft tick, *Ornithodoros moubata,* and the hard tick, *Dermacentor variabilis* [9, 10]. Tick defensins are involved in the tick immune responses with the midgut, hemolymph, and salivary glands. Despite inadequate information dispersed in many tick species, they have served as promising templates for developing new anti-infective agents due to their prominent antimicrobial properties. Extensive microorganisms, especially antibiotic-resistant bacteria and tick-borne pathogens, can be killed by tick defensins. Here we present the general biological characterization of tick defensins with deeply-investigated representatives, shedding light on their function in tick immunity and potential for medical application.

## Methods

This scoping review was conducted adapting the guidelines of Preferred Reporting Items for Systematic Reviews and Meta-Analyses (PRISMA) [11].

### Search strategy

We performed a literature search by employing PubMed and Web of Science databases, covering all published papers in English dated from 2001 to 2022. The databases were explored with the following combination of terms: (tick* OR Ixodidae OR Argasidae) AND (defensin* OR defensin-like OR “antimicrobial peptide*”). We used endnote software for the management of references. The last search was executed on 31 May 2022.

### Inclusion criteria and exclusion criteria

The inclusion criteria involved: studies that reported the discovery and identification of defensins and defensin-like peptides in the tick and studies characterizing the function or mechanism of tick defensins in their original or truncated forms. We excluded annotations for tick defensins in transcriptomic or proteomic analyses that lack further investigation.

### Data extraction and analysis

We classified the articles in line with the source of defensins in tick species. And successive and systematic researches with the same defensin were grouped. The information from common traits to individual features of tick defensins was extracted and summarized.

## Results

**Fig. 1:**
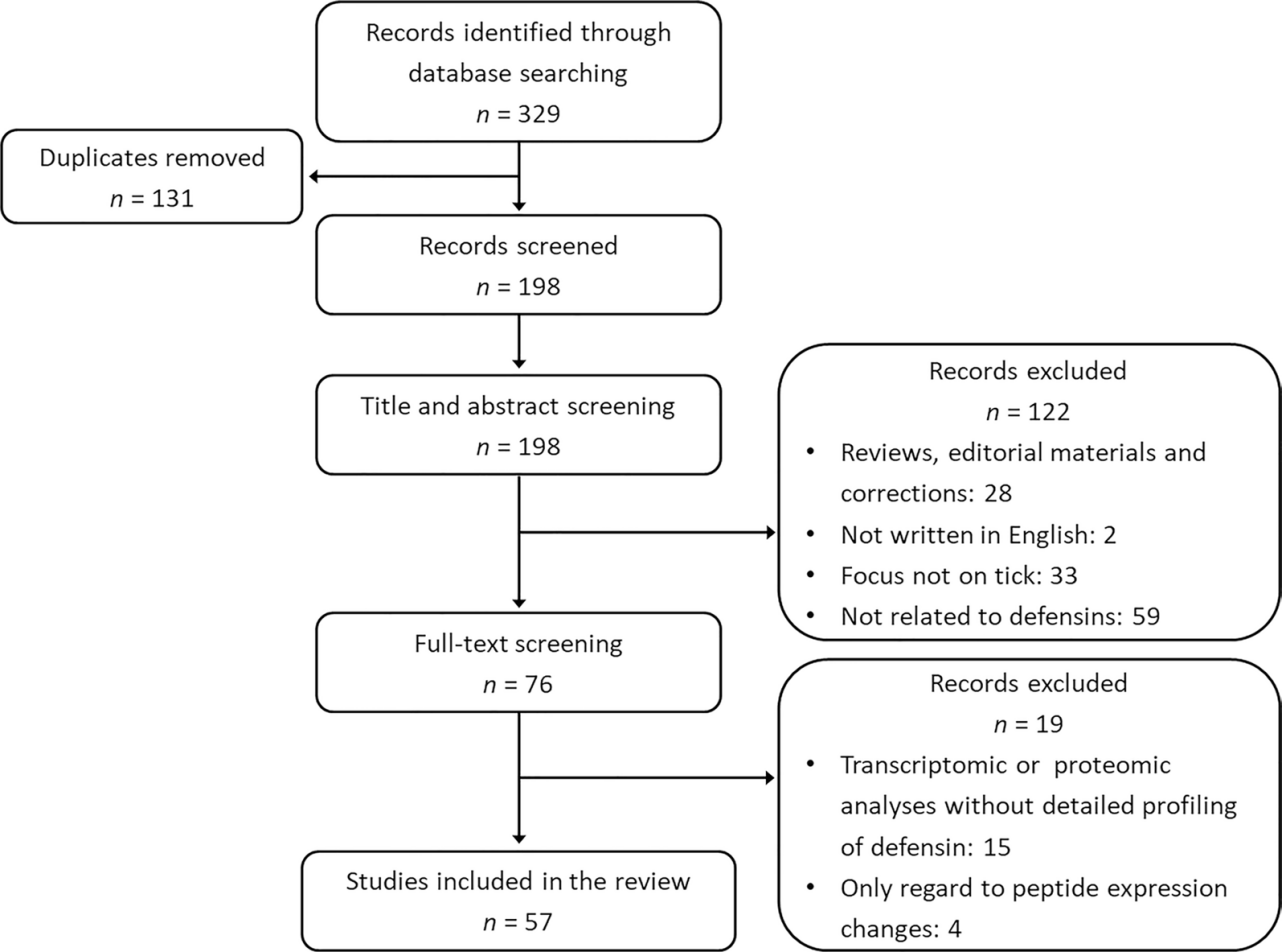
Flow diagram presenting the screening process

Initially, the database searching yielded a total of 329 results, of which 198 articles were screened out after eliminating duplicates. According to the inclusion criteria, 122 and 19 records were discarded through rough screening for title or abstract and thorough assessment for full text, respectively. At last, 57 articles were eligible for our scoping review (Fig. 1).

### Taxonomic status of tick defensins from a structural perspective

Defensins are classified into two evolutionarily independent superfamilies, the *cis*- and *trans*-defensins, by combining primary sequence alignment, secondary structure orientation, disulfide topology, and tertiary structure similarity. Convergent evolution has led to massive structural and functional similarities between these two superfamilies [12, 13]. On the other hand, it has been proposed that *cis*- and *trans*-defensins might share a common ancestor through one amino acid deletion mutation [14]. In the *cis*-defensins, the two most conserved disulfides from the C-terminal β-strand orient in the same direction and bond to the same α-helix. Contrarily, in the *trans*-defensins, the two analogous disulfides point in opposite directions, thus bonding to different secondary structure elements [12, 13]. The presence of disulfide bonds confers a common characteristic to the defensins: high stability to temperature, pH, and proteolysis [13, 15, 16]. Further, the *cis*-defensins are comprised of CSαβ-type (cysteine-stabilized α-helical and β-sheet) defensins from invertebrates, plants, and fungi, whereas *trans*-defensins generally contain vertebrate α-, β-, θ-, and invertebrate big defensins [12].

**Fig. 2:**
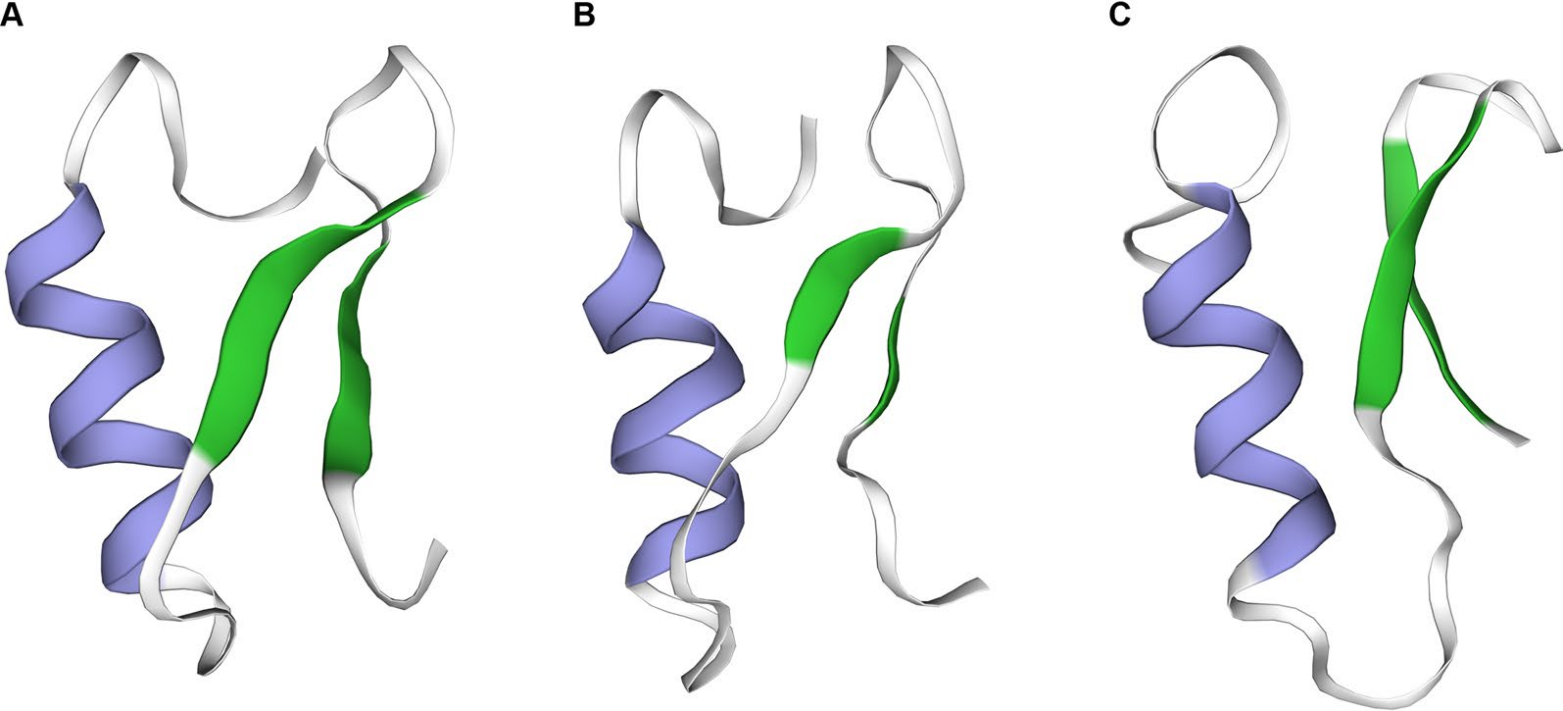
Predicated tertiary structures of representative defensins in **A** *Ixodes ricinus* (AIR77174), **B** *Dermacentor variabilis* (AAO24323), and **C** *Ornithodoros moubata* (BAB41028) based on the template 2Ir5.pdb, 5xa6.pdb, and 2ru0.pdb respectively using the Swiss-Model Protein Modeling Server (http://swiss-model.expasy.org). The α-helixes and the β-sheets are indicated in purple and green

All members of CSαβ-type defensins contain a conserved motif stabilized by disulfide bridges involving one α-helix and one β-sheet of two antiparallel strands. The α helix spanning the CXXXC sequence (X, any amino acid) is anchored to the second β-strand having the CXC sequence via two disulfide bonds. The third disulfide bond links the N-terminal to the first β-strand [17, 18]. Previous studies on the structure–activity relationship have indicated that the functional region of defensins is predominantly located in the C-terminal β-sheet domain, referred to as the γ-core motif [19, 20]. Based on differences in N-terminal sequences, CSαβ-type defensins are grouped into three subclasses: the antibacterial ancient invertebrate-type defensins (AITDs), antibacterial classical insect-type defensins (CITDs), and antifungal plant/insect-type defensins (PITDs) [21]. Commonly, the AITDs have a short n-loop, and a longer n-loop is the feature of CITDs, while the PITDs fold the N-terminal into an extra β-strand [20]. The CITDs are discovered in insects of phylogenetically recent orders. Conversely, the AITDs keep a more distant phylogenetic distribution consisting of primitive insects (e.g., the paleopteran insect *Aeschna* dragonfly), arachnids (spiders, scorpions, and ticks), and bivalvia (mussels and oysters). The PITDs are mainly derived from plants and *Drosophila* [20, 22]. Briefly, tick defensins generally belong to AITDs of CSαβ-type defensins in the *cis*-defensins superfamily [23]. Predicated tertiary structures of three representative tick defensins all have an α-helix at the N terminal and antiparallel β strands to the C-terminal (Fig. 2). Identical with other CSαβ-type defensins, the γ-core motif of tick defensins is functional, which we will discuss later with specific examples.

### Common biological properties of tick defensins

Tick defensins are synthesized as prepropeptides of approximately 8 kDa which generally contain a signal peptide with a highly conserved furin cleavage motif (RVRR) for the release of the mature peptide from the C-terminal [7]. Mature defensins typically consist of about 40 residues featuring six cysteine residues that form three intra-molecular disulfide bridges linked in a Cys1-Cys4, Cys2-Cys5, and Cys3-Cys6 pattern [24]. Most defensins carry a full positive net charge though some anionic peptides have also been reported [25, 26]. Many stimuli related to microbial invasions, such as injury, infection, and blood-feeding, induce the secretion of these protective substances. Defensins were also observed to be up-regulated after molting [27]. A range of tissues in ticks, such as midgut, hemolymph, salivary glands, ovary, and fat body, express defensins, while the distribution of defensins appears to be either tissue-specific or ubiquitous [26]. Tick defensins have a broad spectrum of antimicrobial activity that majorly attack Gram-positive (Gram+) bacteria. Some isoforms are also active against Gram-negative (Gram−) bacteria, fungi, viruses, and protozoa. They universally have low toxicity toward mammalian cells at concentrations that exhibit their antimicrobial effects [28].

**Fig. 3:**
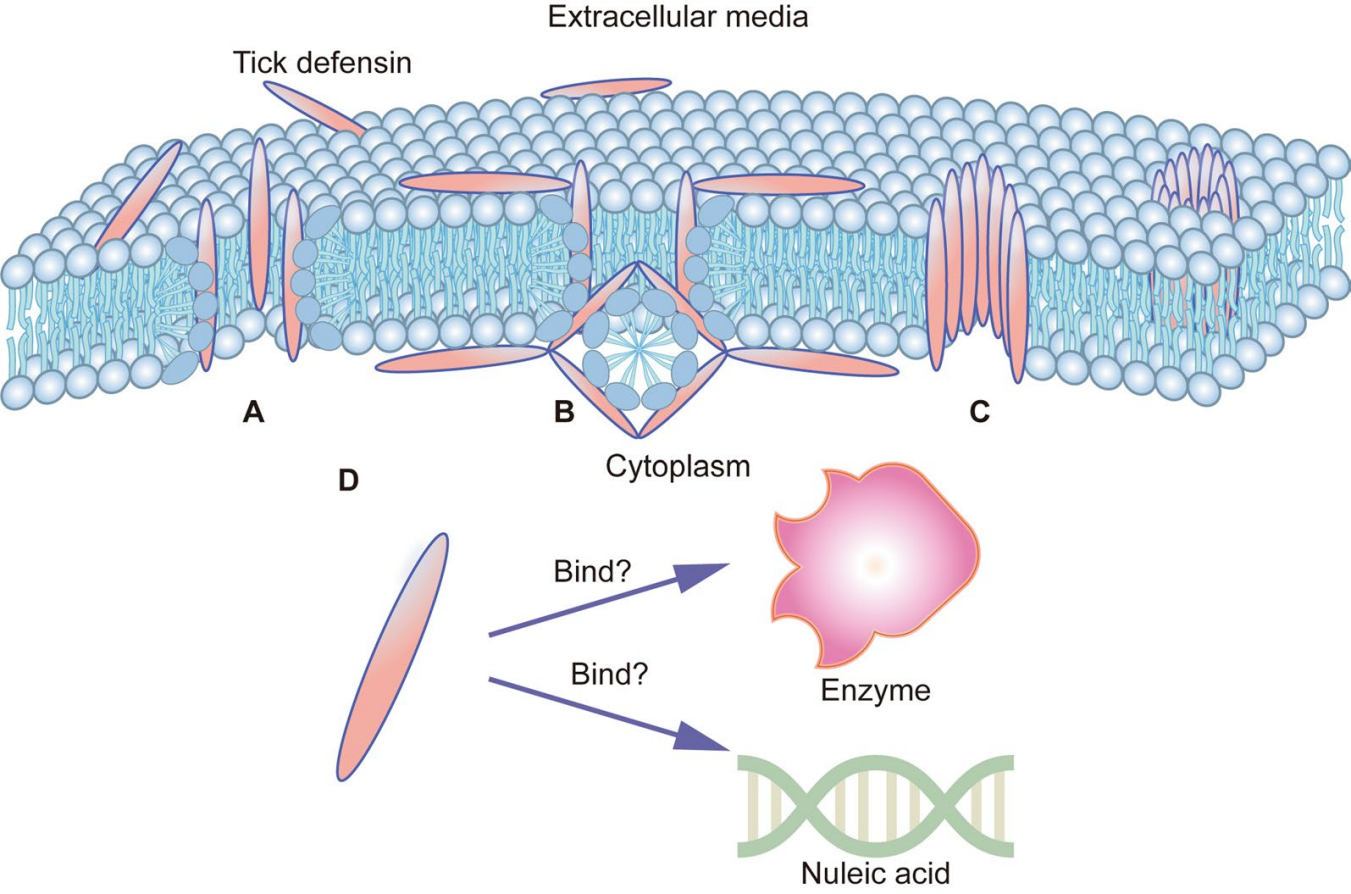
Mode of action of tick defensins. **A**–**C** Similar to other AMPs, the membrane is the primary target of tick defensins. The three typical models proposed to illustrate membrane permeabilization are presented. In the toroidal-pore model (**A**), peptides are inserted between the lipid head groups to form a mixed pore. In the carpet model (**B**), peptides perform as a detergent causing the disruption of bilayer and eventually the formation of micelles. In the barrel-stave model (**C**), peptides aggregate to shape a bundle in the bilayer with a central lumen, like a barrel comprised of peptides as staves. **D** Intracellular components with negative charges like nucleic acids and enzymes are suspected to be the second targets of cationic tick defensins

It is commonly assumed that the antimicrobial activity of cationic AMP is exerted by binding to the negatively charged microbial membranes through electrostatic forces, which leads to the formation of pores in the membranes and subsequently ends in cell lysis. At present, there are at least three generally accepted models used to explain membrane permeabilization: the toroidal-pore model, the carpet model, and the barrel-stave model (Fig. 3) [29]. Similar to these descriptions, the primary action mode of tick defensins is membrane permeabilization. Transmission electron microscopy of defensin-treated *Micrococcus luteus* showed significant lysis of the cytoplasmic membrane resulting in leakage of essential cellular contents [30]. The ability of defensins to penetrate membranes of *Escherichia coli* and *Bacillus subtilis* was observed in studies utilizing fluorescently labeled peptides [31]. Membrane disruption was also revealed in *Toxoplasma* parasites exposed to a potent antimicrobial motif in defensin from hard tick *Haemaphysalis longicornis* [32]. The interaction with peripheral molecules of organisms is a prerequisite to approaching the membrane. For example, the γ-core of the tick defensin DefMT3 was recruited by membrane phospholipids in *Fusarium graminearum,* including phosphatidylserine (POPS), phosphatidic acid (POPA), and phosphatidylglycerol (POPG) [33]. And the active peptides derived from the OsDef2 defensin had a strong affinity towards β-1,3-glucans or mannose residues, the components of cell wall polysaccharides [34]. However, it is speculated that the membrane is not the only target of tick defensin, because multiple anionic targets inside microbial cells like nucleic acids and enzymes may interact with cationic peptides (Fig. 3). The gel retardation assay showed that truncated defensins caused an inhibition of the migration of *E. coli* plasmid DNA (pDNA) at molar charge ratios (pDNA: peptide) close to or above 1:1, suggesting the possibility of the formation of ionic bonds between negatively charged DNA and positively charged peptides [31]. Currently, there are large gaps in the knowledge of mechanisms of tick defensin and further investigations are needed to elucidate whether there are specific membrane receptors and whether there are potential intracellular targets as defensins translocate to the cytoplasm.

**Fig. 4:**
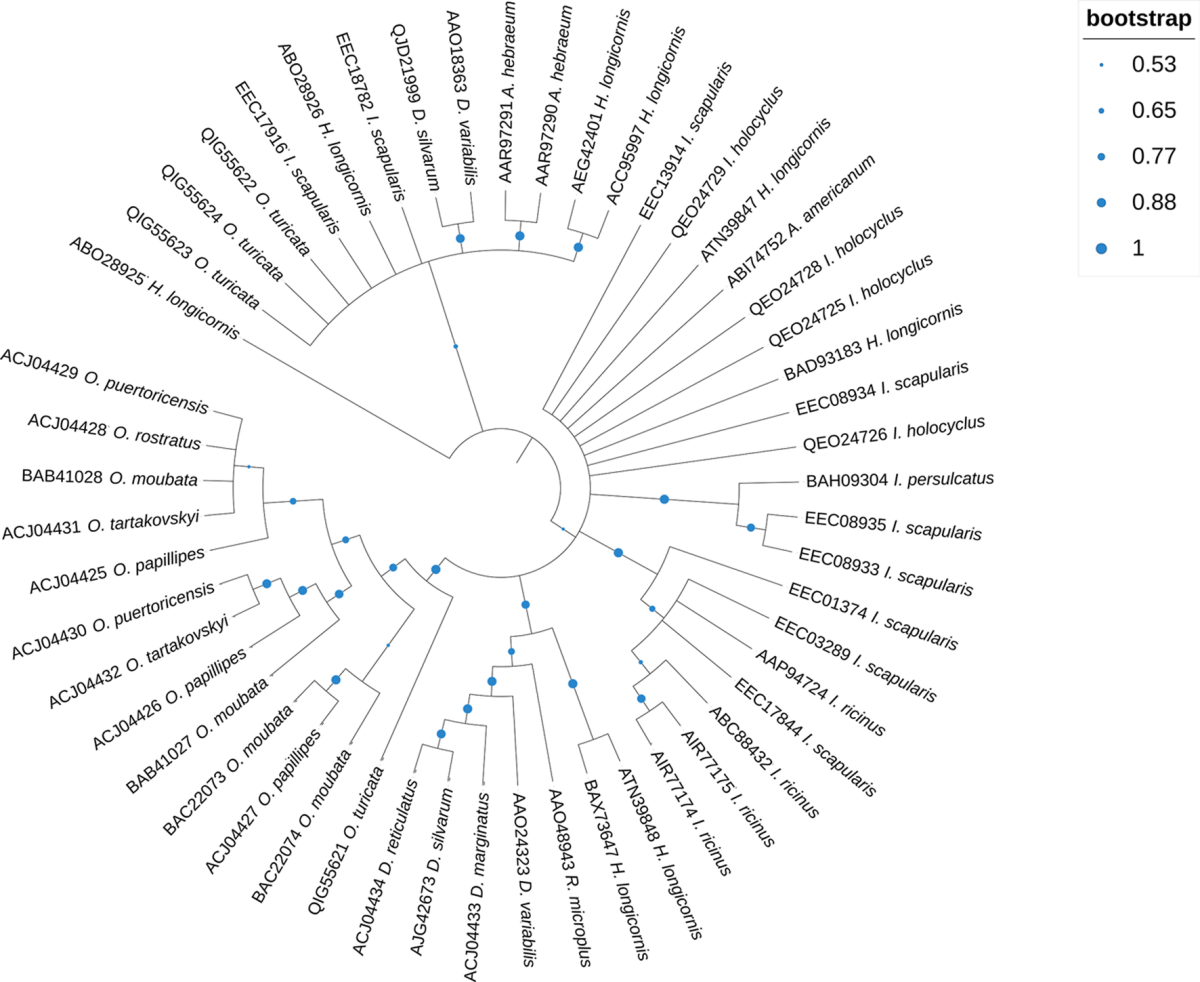
Phylogenetic tree of tick defensins and defensin-like peptides. The evolutionary relationship was assessed using the Neighbor-Joining method in MEGA11 [91]. Genera and accession numbers of each aminoacid sequence used were indicated and bootstrap values over 50% were presented

Defensins and defensin-like peptides have been characterized in many tick genera including *Ornithodoros* in the soft ticks family, *Amblyomma*, *Dermacentor*, *Haemaphysalis*, *Ixodes*, and *Rhipicephalus* in the hard ticks family (Table 1) [9, 35–40]. Multigene families of defensins have been identified in *I*. *scapularis*, *I*. *ricinus,* and *I*. *holocyclus* [23, 26, 41]. Relevant genome and transcriptome datasets have provided abundant and valuable resources for expanding novel defensin families and offered a glimpse of defensin diversity [42–45]. Phylogenetic analysis also indicates a diverse evolutionary history among tick defensins and defensin-like peptides, which formed multiple clades, and defensins within the same tick genus were more likely to cluster together (Fig. 4). Out of considerations for a remarkable degree of variability among tick defensins and coherence of experimental research, well-characterized tick defensins are summarized based on the origin in tick species.

**Table 1:** Summary of tick defensins and defensin-like peptides

Tick families	Tick species	Name	GenBank accession No	References
Argasidae (soft ticks)	*O. moubata*	Defensin A	BAB41028	[9]
	*O. moubata*	Defensin B	BAB41027	
	*O. moubata*	Defensin C	BAC22074	[46]
	*O. moubata*	Defensin D	BAC22073	
	*O. papillipes*	Defensin A	ACJ04425	[35]
	*O. papillipes*	Defensin B	ACJ04426	
	*O. papillipes*	Defensin D	ACJ04427	
	*O. puertoricensis*	Defensin A	ACJ04429	
	*O. puertoricensis*	Defensin B	ACJ04430	
	*O. rostratus*	Defensin A	ACJ04428	
	*O. tartakovskyi*	Defensin A	ACJ04431	
	*O. tartakovskyi*	Defensin B	ACJ04432	
	*O. turicata*	Defensin A	QIG55621	[27]
	*O. turicata*	Defensin B	QIG55622	
	*O. turicata*	Defensin C	QIG55623	
	*O. turicata*	Defensin D	QIG55624	
Ixodidae (hard ticks)	*A. americanum*	Amercin	ABI74752	[36]
	*A. hebraeum*	Defensin protein 1	AAR97290	[25]
	*A. hebraeum*	Defensin protein 2	AAR97291	
	*D. marginatus*	Defensin	ACJ04433	[35]
	*D. reticulatus*	Defensin	ACJ04434	
	*D. silvarum*	Ds-defensin	AJG42673	[38]
	*D. silvarum*	Defensin-like protein	QJD21999	[28]
	*D. variabilis*	Varisin A1	AAO24323	[58]
	*D. variabilis*	Defensin 2	AAO18363	[62]
	*H. longicornis*	Longicin	BAD93183	[37]
	*H. longicornis*	Midgut defensin	ABO28925	[69]
	*H. longicornis*	Salivaryglands defensin	ABO28926	
	*H. longicornis*	Longicornsin	ACC95997	[70]
	*H. longicornis*	Male-specific defensin	AEG42401	[73]
	*H. longicornis*	Hemolymph defensin	BAX73647	[71]
	*H. longicornis*	Defensin DFS1	ATN39847	[74]
	*H. longicornis*	Defensin DFS2	ATN39848	
	*I. holocyclus*	Holosin 1	QEO24725	[41]
	*I. holocyclus*	Holosin 2	QEO24726	
	*I. holocyclus*	Holosin 3	QEO24727	
	*I. holocyclus*	Holosin 4	QEO24728	
	*I. holocyclus*	Holosin 5	QEO24729	
	*I. persulcatus*	Persulcatusin	BAH09304	[75]
	*I. ricinus*	Def1	AAP94724	[80]
	*I. ricinus*	Def2	ABC88432	
	*I. ricinus*	Defensin MT3	AIR77174	[26]
	*I. ricinus*	Defensin MT4	AIR77175	
	*I. scapularis*	Scapularisin-1	EEC08934	[23]
	*I. scapularis*	Scapularisin-3	EEC13914	
	*I. scapularis*	Scapularisin-5	EEC08933	
	*I. scapularis*	Scapularisin-6	EEC08935	
	*I. scapularis*	Scapularisin-16	EEC17916	
	*I. scapularis*	Scapularisin-19	EEC01374	
	*I. scapularis*	Scapularisin-20	EEC17844	[23]
	*I. scapularis*	Scapularisin-22	EEC03289	
	*I. scapularis*	Scasin-1	EEC18782	
	*R. microplus*	Defensin	AAO48943	[40]

*O.*-*Ornithodoros*; *A.*-*Amblyomma*; *D.*-*Dermacentor*; *H.*-*Haemaphysalis*; *I.*-*Ixodes*; *R.-Rhipicephalus*

### Representatives of tick defensins

The family of tick defensins has been expanding since the first tick defensin was isolated in 2001. We pay attention to consecutive and gradually in-depth studies on tick defensins for presenting a thorough introduction and a brief research history of tick defensins.

### Soft ticks defensin

#### *Ornithodoros moubata* defensin

Four defensin isoforms, A–D, divided into two types: defensin A, B, C and defensin D, were identified from the soft tick, *Ornithodoros moubata* [9, 46]. All four defensin genes contained four exons and three introns. The similarity of all four isoforms was more than 78% for the mature portion. Particularly, the mature region of isoforms A, B, and C shared more than 89% homology [46]. All defensin genes were constitutively expressed in entire development stages, providing ticks with rapid resistance against infection. Isoform A, B, and C were expressed at higher levels in eggs and adult females, while D was only elevated in eggs. Tissue specificity showed that defensin A, B, and C were primarily expressed in the midgut, while defensin D expression was strongest in the fat body [47].

Chemically synthetic defensin A presented antibacterial activity against Gram+ bacteria, including pathogenic *Bacillus cereus*, *Enterococcus faecalis,* and methicillin-resistant *Staphylococcus aureus*, but not Gram− bacteria with low hemolytic activity [30]. Furthermore, the involvement of defensins in tick midgut immunity was investigated. Serving as the principal organ of blood meal storage and primary site of tick-pathogen interaction, the midgut is vulnerable to attack from ingested microbes. Moreover, the lack of extracellular digestive proteases and nutritious broth of blood proteins in the midgut lumen makes it a favorable environment for the survival and proliferation of microorganisms. Thus ticks must possess efficient antimicrobial systems in the midgut to eliminate infection or alleviate it to a tolerable degree [5, 7]. Defensin genes were up-regulated following blood-feeding and bacterial injection in the midgut [9, 46, 48]. Correspondingly, a rise in the peptide concentration in response to a blood meal was also demonstrated, and the four mature peptides were detected in the midgut contents from engorged adult females, suggesting the secretion of defensins into tick midgut lumen [46, 49]. The above pieces of evidence confirmed that defensin is a significant component of tick midgut defense.

#### *Ornithodoros savignyi* defensin

In *Ornithodoros savignyi*, two defensin isoforms were found from the midgut designated as OsDef1 and OsDef2 [50]. OsDef2 was used as a template for constructing shorter peptide Os that was derived from the carboxy-terminal domain, and its analog Os-C with three Cys residues removed from the sequence. Both Os and Os-C showed antibacterial activities against both Gram+ and Gram− bacteria, whereas the parent OsDef2 was only active to Gram+ bacteria [50]. A similar result was observed with *O*. *moubata* defensin derivative [51]. In addition to antibacterial activity, Os and Os-C displayed antioxidant, anti-inflammatory, and anti-endotoxin properties, which allowed them to be identified as multifunctional peptides [50, 52].

To minimize active peptides, Os peptide fragments, the overlapping sequences of 10 or 12 residues, were evaluated for antibacterial activity. Os (3–12) and Os (11–22) were screened to be functional against Gram+ and Gram− bacteria. On the other hand, amidation was an approach to improving the antimicrobial ability of shorter derivatives since the enhanced activity of carboxyamidated Os(3–12)NH_2_ and Os(11–22)NH_2_ were manifested [53]. Additionally, Os, Os-C, and Os(11–22)NH_2_ appeared as promising candidates against *Candida albicans* which is an opportunistic fungal pathogen that causes hospital-acquired infection commonly [34, 54]. The candidacidal action was fast-acting and complete killing was finished within 30–60 min at minimum fungicidal concentrations of each peptide ranging from 6 to 28 μmol/L [34].

### Hard ticks defensin

#### *Dermacentor variabilis* defensin

Injection with *Borrelia burgdorferi*, the causative agent of Lyme disease, resulted in discovering a defensin termed varisin from the hemolymph of the American dog tick, *Dermacentor variabilis* [10, 55, 56]. The peptide of varisin was only isolated in the hemolymph or hemocytes, though the transcript was amplified from midgut tissue [57]. The expression of varisin was detected between 15 min and 18 h post-challenge. Defensin was thought to be stored in the hemocytes and released into the hemolymph upon microbial infection [58]. Despite little anti-*Borrelia* activity of varisin alone, its effect was dramatically increased in the presence of chicken lysozyme, reaching more than 65% killing within 1 h, which may be due to a synergistic action between these two peptides [10]. To explore the role of varisin in the immune response, the authors used RNA interference (RNAi) to silence the expression of varisin gene and found that the bacteriostatic activity of the hemolymph was decreased by 2–4 fold, which supported varisin was primarily responsible for the hemolymph defense [59]. In another independent research, the protection of varisin against infection by the Gram− *Anaplasma marginale*, an intracellular pathogen causing bovine anaplasmosis, was studied [60, 61]. However, silencing varisin resulted in a reduction of *A. marginale* infection in male ticks and morphologically abnormal bacteria colonies, which was contrary to the authors' hypothesis [60].

A second defensin isoform, defensin-2, was also described in the hard tick *D. variabilis* which was less than 50% similar to varisin. Compared to the exclusive tissue specificity of varisin, defensin-2 exhibited a universal distribution, including the ovary, midgut, and fat body. When challenged with *Rickettsia montanensis*, the aetiological agent of Rocky Mountain spotted fever, an obvious delay effect on defensin gene transcription was noted in the fat bodies [62]. Moreover, defensin-2 was demonstrated to limit *R. montanensis* infection and associate with the organism leading to cytoplasmic leakage [63].

#### *Haemaphysalis longicornis* defensin

It was discovered that a defensin peptide termed longicin was produced principally in the midgut epithelium from the parasite-bearing tick *Haemaphysalis longicornis*. Along with bactericidal and fungicidal behaviors, longicin remarkably inhibited the proliferation of equine *Babesia equi* at the stage of merozoite, an intra-erythrocytic stage of parasites [37, 64]. Simultaneously, the babesiacidal property was validated in mice infected with murine *B. microti* by reducing parasitemia. RNAi data also revealed the involvement of endogenous longicin in *Babesia* killing and indicated the potential of longicin as a novel agent against zoonotic babesiosis [37]. The researchers also investigated the antimicrobial activity of reduced synthetic analogs from longicin, of which the peptide P4 in the C-terminus attracted their attention. With low cytolytic activity, the efficacy of motif P4 was similar to its full-length parent peptide. The structural analysis of the P4 peptide showed that the antimicrobial activity could be attributed to the β-sheet and the α-helix [37, 65]. Interestingly, the fragment of P4, antimicrobial peptide 1 (AMP1) still retained the anti-babesial function, which was more economical and efficient for chemical synthesis [66]. Further, longicin P4 treatment caused tachyzoites of *Toxoplasma gondii* to lose the capacity to exclude trypan blue dye and to infect the mouse embryonal cell line (NIH/3T3). Longicin P4 bound to tachyzoites and resulted in pore formation, disorganization, and hollowing in the membrane, thus killing the parasites [32]. The functional spectrum of longicin P4 was expanded to Langat virus (LGTV), a naturally attenuated strain as a convenient model of tick-borne flaviviruses [67, 68]. Co-incubation of LGTV with longicin P4 before infection obviously decreased viral foci and virus yield, confirming that the action of longicin P4 against LGTV was through extracellular contact. In contrast to the inactivation of enveloped LGTV, the failure to fight a non-enveloped virus, human adenovirus 25, validated that the virucidal activity of longicin P4 was limited to membrane-coated targets [67].

Apart from the midgut, defensins were detected from a variety of organs in *H. longicornis* comprising the salivary glands [69, 70], hemolymph [71, 72] as well as male accessory glands [73]. Obtained from a cDNA library of *H. longicornis*, two novel defensins were characterized to afford protection for mice threatened by lethal bacterial infection [74].

#### *Ixodes persulcatus* defensin

In the taiga tick, *Ixodes persulcatus*, a defensin named as persulcatusin, was identified that was mainly expressed in the midgut. Persulcatusin was presented as a potent agent to resist the infection of multidrug-resistant *Staphylococcus aureus* strains [75–77]. The significance of the tertiary structure in its combat against bacteria was emphasized as the three-dimensional peptide performed stronger activity than the primary linear one [78].

#### *Ixodes ricinus* defensin

Two defensin isoforms (def1 and def2) were isolated from a cDNA library of *Ixodes ricinus.* Def2 isoform possessed a more potent effect in inhibiting and killing bacteria than def1 [79–81]. Another study identified six novel defensin genes, DefMT2–7, including two isoforms DefMT3 and DefMT4, with different phylogeny and transcriptional expression patterns [26]. Out of the five unique defensins, DefMT3, DefMT5, and DefMT6 had the inhibitory capability to Gram+ bacteria, Gram– bacteria, and fungi. It was noteworthy that the microorganisms attacked by defensins were rather distantly-related, confirming the broad-spectrum role of defensins [82]. Except for DefMT6, DefMT2–7 defensins prevented the growth of apicomplexan parasite *Plasmodium falciparum* in blood stages in vitro. A putative defensin peptide, Scorpions-Ticks Defensins Ancestor (STiDA), was acquired by reconstructing the ancestral amino acid sequence of chelicerate defensins around 444 million years ago, was also functional against *P. falciparum,* which inferred that antiplasmodial capacity of tick defensins had a conserved and ancient characteristic [83]. Furthermore, DefMT2, DefMT5, and DefMT6 significantly reduced rodent-associated *P. chabaudi* parasitemia in a mouse model, suggesting defensin treatment as a new strategy for malaria control [84]. Besides, the γ-core of DefMT3, named TickCore3 (TC3), was a potent antifungal candidate. TC3 efficiently decreased the growth of *Fusarium graminearum* and the production of a severe food toxin from *F. graminearum*. The cysteine cyclization was unnecessary for such capacity, and the positively charged residues, especially lysine 6, exerted an important role in the anti-fungi and anti-mycotoxin features of TC3 [33].

#### *Ixodes scapularis* defensin

Two multigene families of defensins, scapularisin and scasin, with 25 and 21 members respectively, were recognized in the black-legged tick, *Ixodes scapularis,* through comparative genomics approaches [23, 39]. The two multigene families performed high phylogenetic diversity: scapularisins belonged to AITDs, whereas scasins were distantly relevant to AITDs. The γ-core motif of scapularisin-20 was functionally assessed, which displayed antimicrobial potency to Gram+ and Gram− bacteria [23]. Scapularisin-6 was effective against Gram+ *Listeria grayi*. Both scapularisin-6 and scapularisin-3 impeded the germination of phytopathogenic fungi *Fusarium culmorum* and *Fusarium graminearum,* implying their potential for agricultural application [85]. We list the antimicrobial spectrum of several well-investigated tick defensins or defensin motifs in Table 2, which exhibit an extensive range of activity.

**Table 2:** Antimicrobial range of broad-spectrum defensins or defensin motifs

Defensin or defensin motif	Microorganism					References
	Gram+ bacteria	Gram− bacteria	Fungi	Viruses	Protozoa	
*O. savignyi* Os, Os-C and Os(11–22)NH_2_	*Bacillus subtilis*; *Staphylococcus aureus*	*Escherichia coli*; *Pseudomonas aeruginosa*	*Candida albicans*	**–**	**–**	[34, 50, 53]
*D. silvarum* Ds-defensin	*B. pumilus*; *S. aureus*; *Micrococcus luteus*; *Mycobacterium bovis*	*Salmonella typhimurium*; *P. aeruginosa*; *E. coli*	*C. albicans*	**–**	**–**	[38]
*H. longicornis* longicin P4	*S. aureus*	*E. coli*; *P. aeruginosa*; *S. typhimurium*	*Pichia pastoris*	Langat virus	*Babesia* spp. *Toxoplasma gondii*	[32, 37, 67]
*H. longicornis* longicornsin	*S. aureus*	*E. coli*; *P. aeruginosa*; *Helicobacter pylori*	*C. albicans*	**–**	**–**	[70]
*H. longicornis* male-specific defensin	*S. aureus*; *B. licheniformis*	*E. coli*; *P. aeruginosa*; *Serratia rubidaea*; *Psychrobacter faecalis*	*C. albicans*	**–**	**–**	[73]
*H. longicornis* hemolymph defensin	*M. luteus*; *B. cereus*; *S. aureus*	**–**	**–**	Langat virus	**–**	[71, 72]
*H. longicornis* DFS1	*S. aureus*; *M. luteus*; *M. bovis*	*E. coli*; *Borrelia burgdorferi*	*C. albicans*	**–**	**–**	[74]
*I. ricinus* DefMT3 and DefMT5	*Listeria monocytogenes*; *L. fleischmannii*; *L. grayi*; *L. seeligeri*; *S. aureus*	*P. aeruginosa*	*Fusarium culmorum*; *F. graminearum*	**–**	*Plasmodium falciparum*	[82, 83]
*I. ricinus* DefMT6	*L. monocytogenes*; *L. fleischmannii*; *L. grayi*; *L. seeligeri*; *S. aureus*; *S. epidermidis*	*P. aeruginosa*; *E. coli*	*F. culmorum*; *F. graminearum*	**–**	**–**	[82, 83]
*I. holocyclus* holosin 2 and holosin3	*S. aureus*; *S. epidermidis*; *L.grayi*	*E. coli*; *P. aeruginosa*	*F. graminearum*; *C. albicans*	**–**	**–**	[41]

## Discussion

Collectively, tick defensins display a high degree of diversity among soft and hard ticks families or different species within the same genus. This differentiation could be correlated with the distinction of blood-feeding strategies, in which soft ticks tend to feed rapidly (within minutes to hours), whereas the blood meal of hard ticks lasts for several days [7, 35]. The varieties of microorganisms encountered by ticks in their evolutionary process and geographical isolation may also contribute to the diversity of defensins as a selection pressure [35, 83]. Although the investigations of antimicrobial activity of tick defensins are limited, a list of tick-borne pathogens has been targeted, including *Anaplasma*, *Borrelia, Rickettsia,* and protozoa *Babesia*, suggesting the future application of defensins in the treatment of tick-borne diseases. Notably, tick defensins also hold immense potential to deal with the growing number of infectious agents resistant to conventional antibiotics. These peptides have a wide spectrum of antimicrobial activity, a decreased incidence to induce resistance, and the membrane-targeting mode of action, which made peptides not affected by antibiotic-resistance mechanisms [81]. Whether there are other modes of action for tick defensins remains to be further explored. Nonetheless, one of the principal challenges associated with the pharmaceutical application of defensins is the high cost of production. Accordingly, defining the minimum peptides from the parent molecules with retained capacity is an attractive research direction. Several paradigms have been provided, such as the carboxy-terminal fragments from *O. savignyi* and *H. longicornis* defensins and the γ-core motifs from several Ixodes defensins. Further investigations on the clinical applications of these peptides are warranted.

Meanwhile, functional portraits of tick defensins provide some clues about our understanding of tick-pathogen interactions, which influence vector competence, the ability of an arthropod to become a disease vector [86]. Several studies have indicated that defensins play a role in influencing tick vector competence. For example, it was reported that hemolymph of *D. variabilis* incubated with *B. burgdorferi* presented strong borreliacidal activity; however, hemolymph from *B. burgdorferi*-challenged *I. scapularis* did not cause lysis of the bacteria, showing no proof of defensin induction [87]. These results corresponded to the difference in vector competence between *D. variabilis* and *I. scapularis*, in which the former is unable to transmit the bacterium whereas the latter is the natural vector of this pathogen. Moreover, a defensin from tick *D. marginatus* had evident activity against *B. afzelii*, supporting the hypothesis that non-vector ticks could remove *Borrelia* from the body [88]. Besides Lyme disease, the African swine fever virus (ASFV), transmitted by *Ornithodoros* soft ticks, is also an urgent concern. A defensin-like peptide toxin OPTX-1 derived from *O. papillipes* was a competitive inhibitor of ASFV pS273R protease and thus inhibited the replication of ASFV. However, the hard tick-derived defensins displayed much more special inhibitory effects on pS273R protease. The distinct inhibitory efficiencies of tick defensins added the evidence that defensin determines vector competence [89].

Apart from being an essential effector molecule in tick immune response, two tick salivary defensins, IP defensin 1 (IPDef1) and IR defensin 2 (IRDef2) were identified as a novel class of pruritogens in a histamine-independent manner. IPDef1 initiated itch through activating MrgprC11/X1 to sensitize downstream ion channel TRPV1 on dorsal root ganglion neurons. Intriguingly, IPDef1 was also reported to trigger MrgprB2/X2 expressed on mast cells, leading to inflammatory cytokine release and provoking acute inflammation in mice [90]. These new findings extend the function of tick defensins to itch induction, which provides the basis for the precaution and treatment of pruritus caused by the stings or bites of arthropods. As mentioned above, tick defensins contribute to resisting bacterial, fungal, viral, and protozoal infections and influencing tick vector competence in part. In addition, antioxidant, anti-inflammatory, and anti-endotoxin activities were discovered in *O. savignyi* defensins [50, 52]. Together with the role in inducing itch, thus tick defensins can be defined as multifunctional peptides with promising potential in medicine and disease control. More information about the structure–activity relationship is awaited to be explored, which will give us a better understanding of tick defensins.

## Conclusions

As defensins have been found in more tick species, increasing evidence shows that defensins compose an integral class of AMPs in tick innate immunity. Explorations into how defensins react with ingested microbes help us understand the contact between ticks and pathogens, an essential part of tick-host–pathogen interaction. Great advancements have been made in the functional characterization of tick defensins. A large proportion of studies reported their antibacterial, antifungal, and antiprotozoal effects, and few findings described their activities against viruses. In contrast, the resolved three-dimensional structure of tick defensin is absent, which is a limitation for uncovering structural factors influencing biological activity. Determining the minimum functional motif derived from tick defensins will be important for developing tick defensins as therapeutic agents to treat infections.

## Data Availability

The datasets used and/or analysed during the current study are available from the corresponding author on reasonable request.
